# Unilateral Erythema Nodosum following Norethindrone Acetate, Ethinyl Estradiol, and Ferrous Fumarate Combination Therapy

**DOI:** 10.1155/2016/5726416

**Published:** 2016-03-27

**Authors:** Michelle S. Min, Rob Fischer, John B. Fournier

**Affiliations:** ^1^Boston University School of Medicine, Office of Student Affairs, 72 East Concord Street, A2, Boston, MA 02118, USA; ^2^Department of Dermatology, Roger Williams Medical Center, 50 Maude Street, Providence, RI 02908, USA

## Abstract

Erythema nodosum is a septal panniculitis that typically presents as symmetric, tender nodules on the anterior aspects of bilateral lower extremities. Nearly half of cases are due to secondary causes, with oral contraceptive pills being the leading pharmaceutical cause. However, to our knowledge, there has yet to be a published association with norethindrone acetate, ethinyl estradiol, and ferrous fumarate. We report our experience with a 30-year-old woman who developed unilateral tender nodules within a month of starting 1 mg norethindrone acetate and 20 mcg ethinyl estradiol daily. Of note, she had previously taken oral contraceptives with the same estrogen agent but different progesterone, without problems. We conclude that systemically triggered erythema nodosum can present with lesions localized to one extremity. When a patient presents with tender, firm nodules, clinicians should consider the possibility of erythema nodosum and its triggers, such as oral contraceptives. Additionally, should a patient on hormonal therapy develop erythema nodosum, changing the progesterone agent may allow the patient to continue similar therapy without developing symptoms.

## 1. Introduction

Erythema nodosum (EN) is a septal panniculitis that presents as painful, inflammatory nodules or plaques and typically occurs on the anterior aspects of the lower extremities [1]. It occurs in only one to five per 100,000 people. Though it may manifest at any age, it is most prevalent in women in their 20s to 30s [2, 3]. First described in 1798, the etiology and pathogenesis of EN remain unclear today [1]. Most recently, EN has been proposed to be the result of a delayed hypersensitivity reaction in response to a variety of antigens or triggers. Specifically, EN is thought to be a neutrophilic panniculitis caused by immune complex deposits in the venules of the septa of subcutaneous fat [1, 4].

EN is most frequently concluded to be idiopathic (up to 55%), while infections, particularly streptococcal pharyngitis and then tuberculosis, are the most frequently identified associations in adults (28–48%). Sarcoidosis (11–25%), drugs (3–10%), pregnancy (2–5%), and enteropathies including inflammatory bowel disease (1–4%) are other associations found in the literature [2–4]. Interestingly, oral contraceptive pill- (OCP-) related EN has dramatically declined since the 1980s due to the introduction of low-dose hormonal therapy, but OCPs still remain the leading medication associated with EN [2–5]. We describe a young woman who had previously been exposed to OCPs without issues but then developed unilateral EN when exposed to an OCP not yet associated with EN in the literature.

## 2. Case Presentation

**Figure 1:**
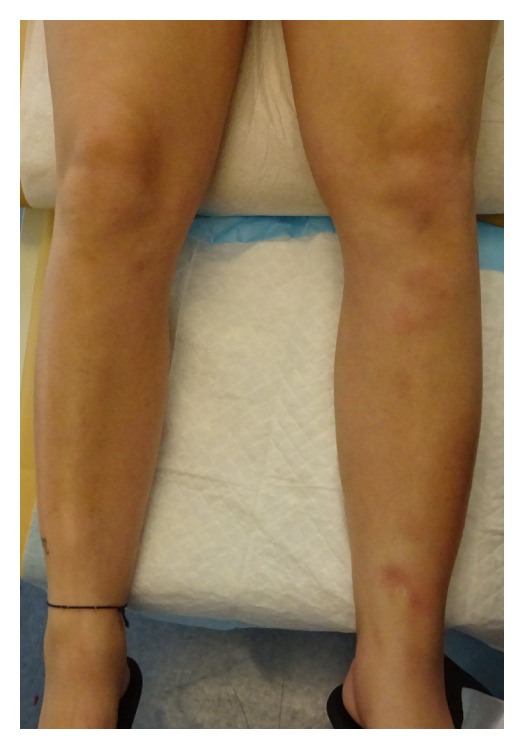
Clinical image depicting violaceous nodules, tender to touch, on the left anterior lower leg.

A 30-year-old woman on daily 1 mg norethindrone acetate, 20 mcg ethinyl estradiol, and ferrous fumarate (Junel Fe 1/20) for 3 weeks presented with a week of tender, dark pink nodules on her left lower leg. She reported developing arthralgias in hips, knees, and ankles 2 weeks earlier and a 3-day fever 1 week prior to the appearance of her lesions. Notably, the patient took 0.18–0.25 mg norgestimate and 35 mcg ethinyl estradiol (Tri-Previfem) without problems 1 year earlier. Physical examination revealed multiple 3–5 cm violaceous, tender nodules, without ulceration, on the left anterior shin and medial malleolus (Figure 1). No lesions were identified on the right leg. White blood cell and platelet counts were slightly elevated at 14,000 cells/cm^3^ and 483,000/microliter, respectively. In order to rule-out other diagnoses, such as erythema migrans or autoimmune disease, Lyme titer and ANA were obtained, which were negative. The patient denied sore throat or cough, so antistreptolysin O titers and chest X-rays were deferred.

**Figure 2:**
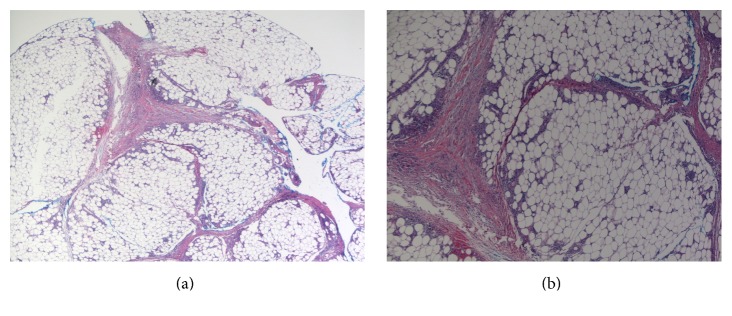
(a) Low-power and (b) medium-power histopathology revealing septal panniculitis and inflammation with septal edema and fibrosis (hematoxylin-eosin stain, 20x and 40x original magnification, resp.).

A double-trephine punch biopsy (4 mm within 1 cm) was performed at the center of a lesion on the left lower leg in order to obtain subcutaneous tissue [6]. Histopathology revealed a septal panniculitis with septal edema and fibrosis, lymphohistiocytic and neutrophilic inflammation, and focal fat necrosis (Figure 2). Findings were primarily identified in the subcutaneous fat. There was no evidence of vasculitis. Periodic acid-Schiff, acid-fast bacilli, and Fite stains were negative for fungal and mycobacterial organisms. A diagnosis of EN was confirmed.

Norethindrone acetate, ethinyl estradiol, and ferrous fumarate OCP combination therapy was discontinued, and naproxen (500 mg orally twice a day) was initiated for symptomatic relief. Within two weeks, lesions began to resolve, and arthralgias improved.

## 3. Discussion

Classically, EN presents as painful, symmetric, erythematous, poorly demarcated but firm nodules varying in size from 1 to 10 cm in diameter. Unlike with vasculitis, lesions tend not to ulcerate. Nodules typically present on the anterior aspects of bilateral legs, but extensor surfaces of forearms, thighs, and trunk may be affected. The few reports of unilateral EN have been associated with ipsilateral infection, including limited tinea pedis and cutaneous leishmaniasis [7, 8]. A prodrome of weight loss, malaise, low-grade fever, cough, and arthralgia may occur 1–3 weeks before. EN tends to self-resolve in 2–6 weeks, usually resolving without scar or atrophy; arthralgias may persist for up to two years [2, 3]. Typically, histopathological examination reveals septal panniculitis with mixed cellular infiltrate of lymphocytes, histiocytes, and giant cells and absence of vasculitis; however, variations of these findings have been described, and repeat biopsy may be necessary when atypical histopathological findings are identified [9].

To the best of our knowledge, there has yet to be a report of EN development in response to norethindrone acetate, ethinyl estradiol, and ferrous fumarate combination (Junel Fe 1/20). This contraceptive is a progesterone-estrogen combination of 1 mg norethindrone acetate (17*α*-ethinyl-19-nortestosterone acetate) and 20 mcg ethinyl estradiol (17*α*-ethinyl-1,3,5(10)-estratriene-3, 17*β*-diol) taken daily for 21 days, followed by 75 mg ferrous fumarate daily for 7 days, for a 28-day regimen [10]. Erythema nodosum is listed as a theoretical risk of this drug due to its classification as an OCP; however this has not yet been explicitly described in literature. Early literature does associate a similar but higher-dose OCP, 5 mg norethynodrel and 0.075 mg ethinyl estradiol, with EN [11].

At first, hormone-related EN was attributed to estrogen rather than progesterone. As more literature emerges regarding the relationship between hormones and EN, this theory seems incomplete. Due to EN favoring the first third of pregnancy, it has been postulated that the ratio of progesterone to estrogen, rather than the quantity of estrogen, is more important [12]. Likely, both estrogen and progesterone form immune complexes or a hypersensitivity reaction [5]. A recent report of a patient who was taking progesterone-only therapy (for assisted-reproductive therapy) and subsequently developed EN further supports the notion that both estrogen and progesterone may have a role in EN [13].

Interestingly, multiple reports have shown that changing the progesterone agent in OCPs may be an option for patients who would like to remain on hormonal contraceptive therapy, regardless of having developed EN [14]. Otherwise, overall treatment for EN involves elimination of the causative agent and initiation of supportive therapy. Bed rest and trauma avoidance to affected regions are recommended. Nonsteroidal anti-inflammatory drugs (NSAIDs) are generally considered first-line treatment for pain management but may be inappropriate in pregnancy due to their mechanism of inhibiting prostanoid activity. Fetal adverse events depend on dosage, duration, and timing of NSAID use during pregnancy. Broadly, NSAIDs increase risk of malformation and miscarriage in early pregnancy and the likelihood of fetal ductus arteriosus premature closure and oligohydramnios in late pregnancy [15]. Alternative therapies include oral potassium iodide 400–900 mg per day and oral prednisone 60 mg daily [2].

We add that unilateral EN in response to a systemic trigger, such as an OCP, is uncommon [7, 8]. Our patient did not have any indications of a local infection limited to the left leg or any anatomic or vascular anomalies to explain the phenomena. However, one might speculate that a minor superficial infection may have preceded EN or have been present in EN's early stages. We cannot definitively make such a conclusion as there were no signs of organisms on biopsy.

Since the introduction of low-dose hormonal therapy, OCP-induced EN is not as commonly reported, but OCPs are still the leading pharmaceutical cause of EN [5]. The patient we described herewith presented with unilateral lesions on her left lower leg, and this development was unique to a particular progesterone-estrogen OCP combination; exposure to the same estrogen but different progesterone was previously well tolerated by the patient. Clinicians should be aware that acute erythema nodosum can present with lesions localized to one leg, and while OCP-induced EN can still occur, changing the progesterone agent in the OCP may allow patients to continue using this form of contraceptive.

## Abbreviations and Acronyms

EN: Erythema nodosum
NSAID: Nonsteroidal anti-inflammatory drug
OCP: Oral contraceptive pill.
